# Immunomodulatory Activity of Dietary Fiber: Arabinoxylan and Mixed-Linked Beta-Glucan Isolated from Barley Show Modest Activities *in Vitro*

**DOI:** 10.3390/ijms12010570

**Published:** 2011-01-18

**Authors:** Anne Berit Samuelsen, Anne Rieder, Stine Grimmer, Terje E. Michaelsen, Svein H. Knutsen

**Affiliations:** 1 Department of Pharmaceutical Chemistry, Pharmacognosy, School of Pharmacy, University of Oslo, Oslo N-0316, Norway; 2 Nofima Mat, Norwegian Institute of Food, Fisheries and Aquaculture Research, Aas N-1430, Norway; E-Mails: anne.rieder@nofima.no (A.R.); stine.grimmer@nofima.no (S.G.); svein.knutsen@nofima.no (S.H.K.); 3 Norwegian Institute of Public Health, Oslo N-0403, Norway, E-Mail: terje.e.michaelsen@fhi.no

**Keywords:** arabinoxylan, mixed-linked β-glucan, barley, Caco-2, complement-fixing test, dietary fiber, HT-29, IL-8, U937, NF-kappaB

## Abstract

High intake of dietary fiber is claimed to protect against development of colorectal cancer. Barley is a rich source of dietary fiber, and possible immunomodulatory effects of barley polysaccharides might explain a potential protective effect. Dietary fiber was isolated by extraction and enzyme treatment. A mixed-linked β-glucan (WSM-TPX, 96.5% β-glucan, Mw 886 kDa), an arabinoxylan (WUM-BS-LA, 96.4% arabinoxylan, Mw 156 kDa), a mixed-linked β-glucan rich fraction containing 10% arabinoxylan (WSM-TP) and an arabinoxylan rich fraction containing 30% mixed-linked β-glucan (WUM-BS) showed no significant effect on IL-8 secretion and proliferation of two intestinal epithelial cell lines, Caco-2 and HT-29, and had no significant effect on the NF-κB activity in the monocytic cell line U937-3κB-LUC. Further enriched arabinoxylan fractions (WUM-BS-LA) from different barley varieties (Tyra, NK96300, SB94897 and CDCGainer) were less active than the mixed-linked β-glucan rich fractions (WSM-TP and WSM-TPX) in the complement-fixing test. The mixed-linked β-glucan rich fraction from NK96300 and CDCGainer showed similar activities as the positive control while mixed-linked β-glucan rich fractions from Tyra and SB94897 were less active. From these results it is concluded that the isolated high molecular weight mixed-linked β-glucans and arabinoxylans from barley show low immunological responses in selected *in vitro* test systems and thus possible anti-colon cancer effects of barley dietary fiber cannot be explained by our observations.

## 1. Introduction

Dietary fiber have been claimed to protect against the development of colorectal cancer (CRC) [1], but according to several reviews, evidence of such a relationship is scarce [2–4]. CRC is one of the most common types of cancer world-wide, and also in Norway, the incidence of CRC has increased over the past 50 years. The reason for this is largely unknown, but lifestyle and diet probably contribute [5,6].

Chronic inflammation is associated with increased risk of cancer development [7], and patients with inflammatory bowel diseases, such as ulcerative colitis and Crohn’s disease, have increased risk of developing CRCs [8]. Plasma levels of the acute phase protein C-Reactive Protein (CRP) which is a marker of inflammation, are elevated in persons who subsequently develop CRC [9]. Increased intake of dietary fiber reduces CRP levels [10–12] as well as the levels of the proinflammatory cytokines IL-6 and TNFα [13]. Strengthening the immune system’s ability to detect and eliminate cancer cells, a process called cancer immunosurveillance [14], on the other hand may have a protective effect. The potential of dietary fiber to promote cancer immunosurveillance is currently unknown. However barley beta-glucan has been shown to increase the effect of anti-tumor antibodies in mice [15,16]. In general, dietary fiber may affect inflammatory processes and immune responses by several mechanisms. Amongst the most studied are the mechanisms exerted by butyrate, a short chain fatty acid produced in the colon following fermentation of dietary fiber. Butyrate has anti-inflammatory [17], apoptotic, and anti-proliferative activities on cancer cells [18,19]. Dietary fiber, depending on their structures, can affect the intestinal immune system by being taken up by M-cells in the Peyer’s patches and transported to underlying immune cells and other cells. This may result in a local cytokine production which can influence T-cells, B-cells, antigen presenting cells and other immune cells. Fiber may also be taken up by intestinal macrophages or dendritic cells (*i.e.*, antigen presenting cells) and transported to lymph nodes, spleen and bone marrow [20,21]. In addition, direct interaction of fiber with colonic epithelial cells or leukocytes may induce changes in immune reactions relevant for inflammation and the development of cancer.

Barley (*Hordeum vulgare*) is an interesting source of dietary fiber and was previously the preferred grain for food in the Nordic region; mainly due to its short growing season due to the climate. In Norway, barley is still the major cereal crop, but only a part is used for human consumption, the majority is used as animal feed. Barley as well as oats are rich in dietary fiber, mainly mixed-linked β-glucans and arabinoxylans [22,23]. In these cereals, β-glucans are linear β-(1→3)/(1→4)-D-glucopyranosyl polymers referred to as mixed- linked or cereal β-glucans [24].

Most of the previous studies on immunomodulatory activities of barley dietary fiber have focused on the mixed-linked β-glucans since they are structurally related to fungal and yeast β-glucans that are β-(1→3)-D-glucopyranosyl polymers with β-(1→6) linked side chains. *In vitro* and *in vivo* experiments on β-glucan preparations from yeast and fungi have shown immunomodulating properties and a potential to increase host resistance against infections [20]. Mixed-linked β-glucans from barley might have similar effects, although knowledge on immunomodulatory effects of barley polysaccharides is quite limited. Some activities have been reported on commercially available barley β-glucan; Intra peritoneal injections of barley β-glucan into fish enhanced the leukocyte count, phagocytic activity, lysozyme activity, complement activity via the alternative pathway and serum bactericidal activity [25]. Czop and Austen [26] found that turbid preparations of barley β-glucan activate the alternative pathway of the complement system *in vitro*. In addition, pre-treatment of human monocytes with barley β-glucan inhibited phagocytosis of zymosan particles [26]. β-glucans enhance cytotoxicity of phagocytes or NK cells towards iC3b-opsonized cells by binding to the lectin site on complement receptor 3 (CR3 or CD11b/CD18, Mac-1, α_M_β_2_ integrin) and thereby initiate cytotoxic degranulation of NK cells and phagocytosis by other cells [27–29]. Oral administered barley β-glucan increased the efficacy of photodynamic therapy of Lewis lung carcinoma in mice through binding to CR3 [27], but barley β-glucan binds to CR3 with lower affinity than yeast β-glucan [29,30]. Barley β-glucans also enhance the anti-tumor effect of monoclonal antibodies in mice when administered orally [16,31] by being taken up by gastrointestinal macrophages, transported to the spleen, lymph nodes and bone marrow where smaller fragments of glucan are bound to CR3 on granulocytes which in turn kill iC3b-opsonized tumor cells [15]. Barley β-glucan can also bind to Dectin-1[32] and activate NF-κB when Dectin-1, Syk, CARD9 and Bcl10 are co-expressed in the cells [33]. The transcription factor NF-κB plays a critical role in immune, cellular stress and inflammatory responses [34].

Instead of using commercially available dietary fiber from barley in the present study we isolated fiber fraction from barley, both mixed-linked β-glucan and arabinoxylan and tested for immunomodulatory activities related to inflammation. This involved extraction and the use of specific hydrolytic enzymes to isolate pure polysaccharide fractions and determination of biological activities by stimulation of the human colon epithelial cell lines Caco-2 and HT-29 followed by measurement of cell proliferation and cytokine secretion. In addition, we investigated the fiber’s ability to modulate NF-κB activity in monocytes and their influence on the complement system using the complement-fixing test [35], all systems involving factors with relevance to inflammatory processes.

## 2. Results and Discussion

### 2.1. Barley Dietary Fiber Fractions

β-glucan and arabinoxylan samples isolated and purified from the common Norwegian barley variety Tyra were the main basis for our investigations. As shown in Table 1, the constituent sugar analysis combined with ^1^H-NMR [23] (spectra not shown) revealed that WUM-BS contained 70% arabinoxylan and 30% mixed-linked β-glucan. Treatment with lichenase (L) and amyloglucosidase (A) efficiently removed most of the remaining mixed-linked β-glucan from this fraction; the enzyme treated fraction WUM-BS-LA contained 96.4% arabinoxylan. Trace amounts of mannose were attributed to the glycoprotein part of the enzyme preparation used. In this fraction, ferulic acid is not present due to alkali treatment during the extraction procedure.

WSM-TP was composed of 90% mixed-linked β-glucan in addition to 10% co-extracted arabinoxylan. Most of the arabinoxylan was removed by enzymatic treatment with xylanase (X). The enzyme treated fraction WSM-TPX was composed of 96.5% glucose and only 3.5% arabinoxylan.

The estimated relative weight average molecular weights (Mw) based on the pullulan series were about 886 and 156 kDa for WSM-TPX and WUM-BS-LA, respectively (Table 1). Molecular weight decreased during the enzymatic treatment of WSM-TP and WUM-BS from about 1090 and 412 kDa, respectively, giving samples with less polydispersities (Mw/Mn).

All previous studies on immunomodulatory activity of mixed-linked β-glucans from barley have been performed on commercially available samples. It should be noticed [20] that choice of isolation method may influence polysaccharide characteristics, such as molecular weight and solubility, and thereby their biological activities. In addition, co-extracted substances or contaminants of an endotoxin nature that may occur during isolation may contribute to significant activities in immunological test systems.

Potential degradation of dietary fiber during food processing has not been taken into account in this study. In addition, the fact that dietary fiber very seldom is eaten alone without subsequent intake of several other food constituents makes the picture quite complex and complicated to explore. In the present study, all fiber fractions had relatively high molecular weight after isolation, and no attempts were made to alter the chain length in either of the samples. This was because we primarily wanted to investigate intact carbohydrate dietary fibers with the presumption that dietary fiber remains undegraded until reaching the microflora in the colon.

### 2.2. Effect on IL-8 Secretion and Cell Proliferation in Caco-2 and HT-29

To test the inflammatory response of the fiber fractions on gut epithelial cells the modulation of IL-8 (CXCL8) secretion from the human intestinal epithelial cell lines Caco-2 and HT-29 cells was determined. The concentration used of 1 mg/mL is physiologically relevant as a concentration of 1 mg/mL barley fiber in the intestine corresponds to the consumption of approximately 20 g barley, an amount found, for example, in two slices of barley bread (40% barley flour). In addition, the potential toxic effect of the fiber fractions on the Caco-2 and HT-9 cells was determined by measuring the effect of the fiber fractions at different concentrations on cell proliferation using the MTT assay.

**Figure 1:**
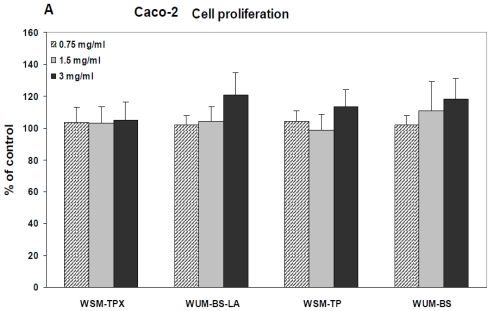

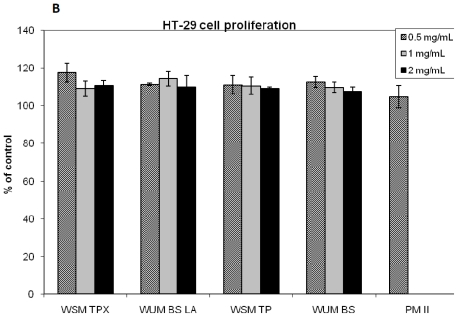
The effect of fiber fractions extracted from the barley variety Tyra on cell proliferation of (**A**) Caco-2 cells and (**B**) HT-29 cells. Cells were incubated with three different concentrations of the respective fiber fraction in cell culture medium for 24 hours before cell proliferation was measured. Each bar represents the mean of at least three experiments performed in triplicate (as % of medium control) ± SD.

**Figure 2:**
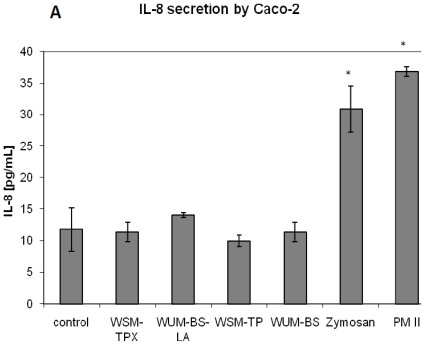

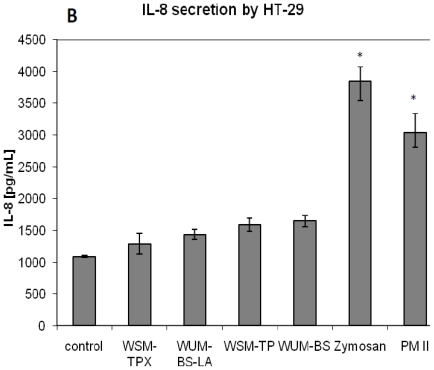
IL-8 secretion from Caco-2 (**A**) and HT-29 (**B**) cells in response to treatment with fiber fractions extracted from the barley variety Tyra, zymosan and PMII (all 1 mg/mL). Cells were incubated with fiber of the respective fiber fractions in cell culture medium for 24 hours before IL-8 secretion was measured. Each bar represents the average ± SD of one representative experiment from a total of three independent experiments. * *p* < 0.05.

We found that the fiber fractions had no significant effect on the cell proliferation of the human intestinal epithelial cell lines Caco-2 and HT-29 cells (Figure 1A, B). It was observed that the HT-29 cell line in general secretes considerably higher levels of IL-8 than Caco-2, but the barley fiber fractions had no significant effect on this secretion either from Caco-2 (Figure 2A) or HT-29 cells (Figure 2B). Only the positive controls, PMII and zymosan increased secretion of IL-8 from both cell lines significantly (*p* = 0.001) compared to the respective controls. Zymosan is a crude extract from yeast (*Saccharomyces cerevisiae*) and contains mainly β-glucan but also some mannan [20], protein, fat and chitin [36]. Immunomodulatory activities of β-glucan from yeast and from other sources have been studied extensively, for review on this topic see [37]. PMII is a pectic polysaccharide fraction isolated from *Plantago major* L. leaves, a plant used in traditional medicine to aid the healing of wounds. PMII has shown immunomodulatory activities both *in vitro* and *in vivo*: Increased resistance against bacterial infection in mice, activation of human monocytes and activation of the complement system [35,38,39]. PMII is therefore considered useful as positive control in immunological test systems.

Even though we did not find any direct effect of the barley fiber fractions on the intestinal epithelial cell model system, barley fiber may affect inflammatory processes and immune response by other mechanisms. As outlined in the introduction, barley fiber may be taken up by intestinal macrophages or M-cells and delivered to underlying immune cells where binding of barley β-glucan to the lectin site of CR3 on effector cells has been shown to enhance cytotoxic activity [15,27].

### 2.3. The Ability of the Fiber Fractions to Modulate NF-κB Activity in Monocytes

**Figure 3:**
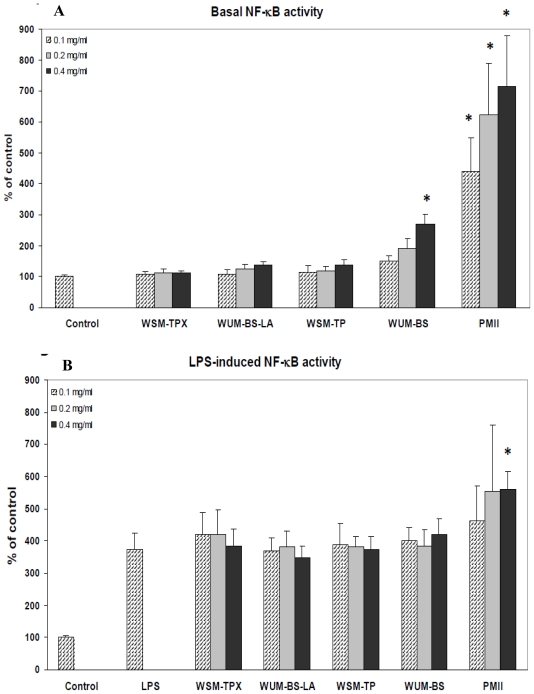
The effect of the fiber fractions extracted from the barley variety Tyra on basal (**A**) and LPS-induced (**B**) NF-κB activity. U937-3xκB-LUC cells were incubated with 0.1, 0.2 or 0.4 mg/mL as indicated of the respective fiber fraction in cell culture medium for 6.5 hours before luciferase activity was measured. For LPS-induction, 1 μg/mL LPS was added after 30 min, and the cells incubated further for six hours before the luciferase activity was measured. Each bar represents the mean of at least three experiments performed in triplicate ± SD. * *p* < 0.05.

The nuclear transcription factor kappa B (NF-κB) plays a central role in inflammatory response [40]. Thus, to further study the effect of the fiber fractions on the immune response, the ability of the fiber fractions to modulate basal and LPS-induced NF-κB activity was tested using the U937-3κB-LUC monocytic cell line stably transfected with a luciferase reporter containing three NF-κB binding sites. It has been shown that this model system correlates well with *in vivo* NF-κB activity [41,42] Due to the limitations of the test system, lower concentrations of the samples (0.1, 0.2 and 0.4 mg/mL) were used compared to experiments with the Caco-2 and HT-29 cell lines. However, as the U937-3κB-LUC cell line is quite sensitive, the response is still considered relevant. The activities of the different fiber fractions were compared to the positive control, PMII [39]. Of the different fiber fractions only the highest concentration of WUM-BS had a significant effect on basal NF-κB activity (*p =* 0.004) (Figure 3A), giving an increase of the activity to 270% compared to control. The apparent dose response from 0.1 mg/mL to 0.4 mg/mL of all fractions of the basal NF-κB activity was statistically not significant compared to the control. However, all concentrations of PMII significantly increased basal NF-κB activity (*p* < 0.001) in the test system compared to the control. None of the fiber fractions had significant effect on the LPS-induced NF-κB activity, only the highest concentration of PMII increased the LPS-induced NF-κB activity significantly (*p* = 0.013) (Figure 3B).

Only high concentrations of a fraction containing 70% arabinoxylan and 30% mixed-linked β-glucan (WUM-BS) increased the activity of the pro-inflammatory transcription factor NF-κB in monocytes. This fraction was obtained after alkaline extraction of a water insoluble residue. Neither pure arabinoxylan nor pure mixed-linked β-glucan was active in this test system. Some biological effects of β-glucans are initiated by binding to Dectin-1 on macrophages and dendritic cells. Barley β-glucan has previously been found to activate NF-κB when Dectin-1, Syk, SARD9 and Bcl10 were co-expressed in the cells [33], and it was concluded that Dectin-1 was involved in these activities. However, binding to Dectin-1 requires β-glucans with a minimum of 10- or 11- mer 1,3-linked glucose oligomers [43] which are structural elements not found in barley. Barley β-glucans only contain single 1,3-linked glucose units separating two or three 1,4-linked glucose oligomers [44]. Transcription factor NF-κB can be activated via many different pathways including proinflammatory cytokines, TLR activation, for example, by LPS and by T-cell activation [40]. As shown in Figure 3B, LPS induced NF-κB activity was not significantly altered by any of the barley fractions indicating that the basal activity observed after stimulation of 0.4 mg/mL WUM-BS may be due to contamination by LPS. In any case, the activity found in Figure 3A cannot be due to either arabinoxylan or mixed-linked β-glucan since other fractions containing higher levels of mixed-linked β-glucan (WSM-TP and WSM-TPX) and arabinoxylan (WUM-BS-LA) were inactive in the test system. The positive control PMII increased LPS induced activity but to a lesser extent than measured with PMII alone (Figure 3A). This shows that PMII is active *per se*, but confirms presence of LPS. Previously, it has been shown that PMII can activate monocytes and induce secretion of TNFα [39]. One might speculate that secreted TNFα in turn activate NF-κB [40], alternatively PMII may bind to NF-κB activating receptors directly.

### 2.4. Complement Fixing Test

**Figure 4:**
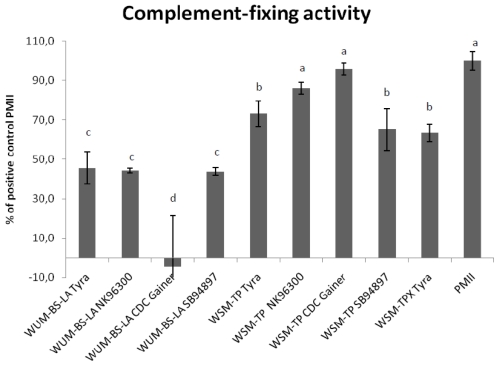
Complement fixing test of fiber fractions isolated from barley varieties. Each bar represents % activity (mean values of triplicates ± SD) of the positive control PMII measured at 1 mg/mL. The fractions are arabinoxylan (WUM-BS-LA) and mixed-linked β-glucan rich fractions (WSM-TP and WSM-TPX) from the barley varieties Tyra, NK96300, SB94897 and CDC Gainer. Activity bars denoted with the same letter (a, b or c) are not significantly different, *p* < 0.05.

Purified β-glucan (WSM-TPX) and arabinoxylan (WUM-BS-LA) from Tyra were tested for activity in the complement-fixing test. Both showed lower activities than the positive control, PMII. At 1 mg/mL WSM-TPX was significantly more active than WUM-BS-LA (*p* = 0.009) (Figure 4).

β-glucans and purified arabinoxylans from other barley varieties [45] were also subjected to this test. As shown in Figure 4, all arabinoxylan fractions (WUM-BS-LA) had relatively low activity compared to the positive control. Arabinoxylan isolated from Tyra, NK96300 and SB94897 had very similar activities; the one from CDC Gainer was almost inactive.

Starch-free mixed-linked β-glucans that had not been subjected to a xylanase treatment (WSM-TP) containing additional small amounts of arabinoxylan, had the highest activity in this test system. Such WSM-TP samples originating from CDC Gainer and NK96300 showed activity at the same level as the positive control, while similar fractions from Tyra and SB94897 were less active (*p* < 0.035). Figure 4 furthermore shows that a xylanase purification step of WSM-TP into purified mixed-linked β-glucan (WSM-TPX) did not alter the complement-fixing activity significantly.

In general, all mixed-linked β-glucan rich fractions had a significantly higher complement-fixing activity than the arabinoxylan-rich fractions (*p* < 0.027).

According to Figure 4 the fractions can be listed as follows with regard to decreasing activity in the complement fixing test: PMII = WSM-TP CDC Gainer = WSM-TP NK96300 > WSM-TP Tyra = WSM TPX Tyra = WSM TP SB94897 > WUM BS-LA Tyra = WUM-BS-LA NK96300 = WUM-BS-LA SB94897 > WUM-BS-LA CDC Gainer.

Mixed-linked β-glucans from barley have previously shown to activate the complement system via the alternative pathway [26], the present study demonstrates an effect also on the classical pathway. The complement system provides a first line of protection against potential harmful invaders and is part of the innate immune system. It consists of a group of serum proteins that are activated in a cascade mechanism. Many of these proteins are pro-enzymes that are activated by proteolytical cleavage which in turn activate the next step in the cascade. Activation can be initiated by three pathways; the classical pathway, the alternative pathway or the lectin pathway, and is important for initiating inflammation, activation of leucocytes, lysis of target cells and opsonisation [46,47]. The test system employed has some limitations since it does not distinguish between activation and inhibition of the complement cascade, only a “consumption” of complement activity is registered. From previous studies however, it is established that PMII, the positive control, is an activator of the complement system [35], and it has also shown to protect against bacterial infection *in vivo* [38].

The mixed-linked β-glucan fractions tested were more active than the arabinoxylan fractions. The reason for the differences in activity of the different β-glucans might be due to differences in their primary structure. The ratio of (1→4)/(1→3) linkages present varies between the different barley varieties tested. NK96300 has the highest ratio (2.76) followed by CDC Gainer (2.59), Tyra (2.48) and SB94897 (2.30) [23]. The varieties with the highest (1→4)/(1→3) ratio have the highest activity in the complement fixing test, but statistical analysis shows no significant correlation between linkage ratio and activity or between molecular weight of the WSM-TP fractions and activity. Mw of WSM-TP fractions from NK96300, CDC Gainer and SB94897 were estimated to 1040, 1130 and 1040 kDa, respectively. The estimated molecular weights of the WSM-TP fractions were significantly higher than the corresponding arabinoxylan (WUM-BS-LA) fractions (*p* < 0.001). Mw of the WUM-BS-LA fractions from NK96300, CDC Gainer and SB94897 were estimated to 214, 203 and 190 kDa, respectively. The WUM-BS-LA fractions from different barley varieties tested had in general low activities in the complement fixing test. However, when both these arabinoxylan fractions and the more active β-glucan rich fractions were included in the statistical test a significant positive correlation was found between molecular weight and activity (*p =* 0.002). On the other hand, when these two classes of dietary fiber are evaluated separately there is no correlation between activity and their estimated molecular weights. Contamination of LPS does not affect this test system [48], so activity is not attributed to presence of endotoxin.

The arabinoxylans from different barley varieties tested had in general low activities in the complement fixing test leaving this hemicellulose type of dietary fiber non-responsive in all the test systems in the present study. To our knowledge, high molecular weight arabinoxylans have not been ascribed immunomodulatory activities. On the other hand arabinoxylan oligosaccharides have been studied for prebiotic properties [49] and have been shown to reduce preneoplastic lesions in the colon of rats treated with a carcinogen [50].

## 3. Experimental Section

### 3.1. Isolation of Fiber Fractions

Fiber fractions were extracted from four barley varieties; NK96300, Tyra, CDC Gainer and SB94897 basically as previously described [51]. Briefly; milled (0.5 mm) barley samples (48 g) were extracted and washed with boiling ethanol. This removed low molecular weight constituents and is promoting the denaturation of endogenous hydrolytic enzymes such as β-glucanase. Following defatting with hexane, extraction with boiling water gave a water soluble material (WSM) and a residue of water insoluble material (WUM). WSM was furthermore treated with 7 mL amylase (Termamyl 120 L, Type L, Novozymes ) and 75 mg protease (Porcine Pancreatine, SIGMA) filtered and recovered with alcohol precipitation resulting in the starch free fraction designated WSM-TP.

In an attempt to remove small amounts of co-extracted arabinoxylan, WSM-TP was treated with a xylanase. WSM-TP Tyra (1 g) was dissolved in sodium acetate buffer pH 4.5, and 10 μL (21 U) *endo* β-xylanase (β-xylanase M6, Megazyme) was added at 40 °C and left for 3 h with gentle stirring. Polysaccharide material was precipitated with isopropanol and centrifugated at 1000 × g for 10 min. The pellet was redissolved in water, dialyzed against distilled water using a dialyzing tube with cut off 12,000–14,000 (Medicell Int. Ltd); freeze dried and designated WSM-TPX.

Base soluble material (WUM-BS) was then extracted from the previous water insoluble residue (WUM) with 1 M NaOH added 1% NaBH_4_. Co-extracted mixed-linked β-glucans and starch were removed by adding 50 U lichenase (Lichenase EC 3.2.1.73, Megazyme) and 400 μL amyloglucosidase (Amyloglucosidase for Total Dietary Fiber Assay EC 3.2.1.3, SIGMA) as described elsewhere [51] giving fractions designated WUM-BS-LA.

### 3.2. Monosaccharide Composition

Methanolysis combined with TMS-derivatisation and GC were performed according to the method of Chambers & Clamp [52] with modifications as previously described [53] using 4 M HCl in anhydrous methanol for 24 h at 80 °C.

### 3.3. ^1^H-NMR

^1^H-NMR spectra of selected samples were obtained on a Varian Mercury 300 system. Approximately 4 mg of freeze dried material was solubilized in 0.7 mL D_2_O, transferred to NMR glass tubes and acquired at 80 °C with typically 64 scans. Further details of the method are described in Knutsen & Holtekjolen [23].

### 3.4. HPLC

GPC-SEC was performed using a DIONEX P680 pump with a Spectraphysics AS3500 auto injector and a Shimadzu RID6A refractive index detector controlled with Chromeleon 6.80 software. Serially connected Shodex OHPack SB-806-HQ and SB 804-HQ columns were connected to a Shodex OHPack SB-LG precolumn and eluted at 40 °C with 50 mM Na_2_SO_4_ (0.5 mL/min), and samples (1 mg/mL) were injected using a 100 μL loop. Relative molecular weight averages (Mw and Mn) were estimated offline by the software WINGPC −6.2 using pullulan molecular weight standards ranging from Mp 342 to 1,520,000 Da for calibration. Software and standards were obtained from PSS (Polymer Standards Service GmbH, Mainz, Germany).

### 3.5. Cell Cultures

The Caco-2 cell line (obtained from the American Type Culture Collection (ATCC), and a generous gift from Professor Kirsten Sandvig, Norwegian Radium Hospital) and HT-29 cell line (obtained from ATCC, and a generous gift from Professor Tor Lea, Norwegian University of Life Sciences) were grown in DMEM medium containing 10% fetal calf serum, 1% non-essential amino acids, 100 U/mL penicillin, and 100 mg/mL streptomycin. The U937-3xkB-LUC cell line (a generous gift from Professor Rune Blomhoff, University of Oslo) was grown in RPMI-1640 medium supplemented with 10% fetal bovine serum, 2 mM L-glutamine, 50 U/mL penicillin, 50 mg/mL streptomycin and 75 μg/mL hygromycin (Sigma-Aldrich, St. Louis, MO). The cells were maintained at 37 °C and 5% CO_2_ in a humidified incubator. If not otherwise stated, all solutions were obtained from Invitrogen (Carlstad, CA).

### 3.6. Measurement of Cell Proliferation and IL-8 Secretion

For IL-8 secretion, cells were plated in 12-well plates. For cell proliferation, cells were plated in 96-well plates. Cells were plated at a concentration of 1.0 × 10^5^ cells/mL (Caco-2) and 1.5 × 10^5^ cells/mL (HT-29) and incubated until they reached 80 % confluency (48 h). The fiber fractions were solubilized in water by boiling for 20 min, aliquoted, freeze dried and re-solubilized in growth medium to treatment concentrations of 0.5–3 mg/mL. A yeast derived beta-glucan (Zymosan A Z4250 Sigma) and PMII, a plant polysaccharide fraction from *Plantago major* L with known immune stimulating activity [35,38,39] were used as positive controls for IL-8 secretion. Cells were incubated 24 h with 1.5 mL (12-well plates) or 100 μL (96-well plates) growth medium or solubilized fiber fractions in duplicate (12-well plates) or triplicate (96-well plates). At the end of the incubation, the plates were processed for measurement of either cell proliferation or IL-8 secretion.

Cell proliferation was determined using the colorimetric MTT assay (Roche Diagnostics GmbH, Mannheim, Germany) that measures the ability of metabolic active cells to cleave tetrazolium sodium salt to purple formazan crystals [54]. The resulting purple precipitate in each cell was dissolved in 100 μL isopropanol containing 0.04 M HCl, and the absorbance measured at 562 nm using Titertek Multiscan plus MK II plate reader (Labsystems, Finland). IL-8 concentrations in the cell culture supernatants were determined using an enzyme linked immunosorbent assay (ELISA). Monoclonal mouse anti-human IL-8 antibody (BD Bioscience Pharmingen, San Diego, CA) suspended in coating buffer (0.1 M Carbonate/Bicarbonate buffer pH 9.6) was added to MaxiSorp^TM^ ELISA plates (Nunc, Roskilde, Denmark) and incubated over night at 4 °C. Plates were washed three times with PBS containing 0.01% Tween-20 and unspecific binding-sites were blocked by incubating with 5% BSA in PBS for 1 h at room temperature. After washing five times with PBS-Tween, samples and human recombinant IL-8 standards (BD Bioscience Pharmingen,) diluted in working strength high performance ELISA (HPE) buffer from Sanquin (Amsterdam, Netherlands) were added to the plates, which were then incubated for 1.5 h at room temperature followed by washing five times with PBS-Tween. Plates were then incubated for 1 h with biotinylated mouse anti-human IL-8 monoclonal antibody (BD Bioscience Pharmingen) in HPE buffer. After another washing step streptavidin-horseradishperoxidase conjugate (BD Bioscience Pharmingen) in HPE buffer was added and incubated at room temperature for 30 min. Plates were then washed five times with 30 sec between each wash. Color developed after addition of 3,3′,5,5′-tetramethylbenzidine (Sigma-Aldrich) in 0.05 M Phosphate-Citrate-Buffer containing H_2_O_2_. After 10 min the reaction was stopped by addition of 1 M H_2_SO_4_, and absorbance was measured at 450 nm using the Titertek Multiscan plus MK II plate reader (Labsystems, Finland). The detection limit of the IL-8 ELISA was 2 pg/mL.

### 3.7. NF-kB Activity Assay

In order to measure NF-κB activity the U937-3xκB-LUC cell line were transferred to RPMI medium with 2 % fetal bovine serum and seeded out in 96 well plates. The fiber fractions extracted from the barley variety Tyra were mixed with highly purified water (Milli-Q, 18.2 MΩ) to a final concentration of 1 mg/mL in Precellys CK14 homogenization tubes (Bertin Technologies, Montigny le Bretonneux, France), solubilized using the Precellys 24 homogenizer (Bertin Technologies) followed by boiling the samples for 5 min, and then freeze dried. The resulting freeze dried fiber fractions were dissolved in medium with 2% serum to a concentration of 4 mg/mL. PMII from *Plantago major* L. leaves was used as positive control [35,38,39]. This final solution of the fiber fractions was diluted directly in the wells giving the final concentrations of 0.1, 0.2 and 0.4 mg/mL. To measure basal NF-κB activity, cells were incubated with fiber fractions or vehicle control for 6.5 hours. To measure lipopolysaccharide (LPS)-induced NF-κB activity, cells were pre-incubated with fiber fractions or vehicle control for 30 min, then 1 μg/mL lipopolysaccharide isolated from *E. coli* 0111:B4 (Sigma-Aldrich, St. Louis, MO, U.S.) was added to the cells and the incubation continued for 6 hours. Cell viability for these cells was determined with the use of the CellTiter-Glo Luminiscent Cell Viability Assay (Promega, Madison, WI, U.S.) with cut-off value of 10% non-viable cells. The NF-κB activity was determined by measuring the luciferase activity after addition of Bright-Glo^TM^ Reagent (Pomega, Madison, WI, U.S.) in accordance to the manufacturer’s instructions. Luminescence was detected for 1 sec using the Glomax96 Microplate Luminometer (Promega, Madison, WI, U.S.).

### 3.8. Complement Fixing Test

Human complement proteins were incubated with fiber fractions that might either activate or inhibit activation of the complement proteins. In both situations complement activity is depleted with a negative influence on a balanced hemolysis system involving antibody-sensitized sheep red blood cells and a human serum diluted to give 50% hemolysis. The degree of hemolysis was measured as absorbency at 405 nm. Fiber fractions were tested in triplicates using PMII, a polysaccharide fraction from *Plantago major* L. as positive control [35,39].

### 3.9. Statistics

Analysis of significant differences was tested by one-way analysis of variance (ANOVA) with Dunnett’s comparisons with a control and Pearson correlation analysis using Minitab Version 16. Differences were considered significant when *p* < 0.05.

## 4. Conclusions

From the experiments presented, it is concluded that purified high molecular weight mixed-linked β-glucans from barley have quite low immunological responses and do not affect proliferation and secretion of IL-8 of the colon epithelial cell lines Caco-2 and HT-29, or NF-kappaB activity in the monocytic cell line U937-3κB-LUC but are active in the complement-fixing test. High molecular weight barley arabinoxylans have neglectible activities in all test systems mentioned. Taken together the results do not support that barley dietary fiber protect against the development of CRC through the immune responses or inflammatory responses tested. Still, one cannot overrule that such effects may occur through other mechanisms that may be shown in other test systems.

## Figures and Tables

**Table 1:** Characterization of fiber fractions from *Hordeum vulgare* var. Tyra: monosaccharide composition (mol %) and estimated molecular weight calculated on the basis of pullulan standards by GPC-SEC and refractive index detection, as weight average (Mw) and number average (Mn) of barley fiber fractions.

	Monosaccharide Composition (mol%)				Molecular Weight (kDa)	
Fraction	Glc	Man	Xyl	Ara	Mw	Mn
WSM-TP	91.0	0.0	5.8	3.3	1090	599
WSM-TPX	96.5	0.0	1.9	1.6	886	501
WUM-BS	30.0	0.0	52.7	17.3	412	98
WUM-BS-LA	2.0	1.6	66.0	30.4	156	42

## References

[1] Burkitt DP Some diseases characteristic of modern westernised civilisation Br. Med. J 1973 1 274 278 4568142 10.1136/bmj.1.5848.274 PMC1588096

[2] Asano TK McLeod RS Dietary fibre for the prevention of colorectal adenomas and carcinomas Cochrane Database Syst Rev 2002 1 CD003430 12076480 10.1002/14651858.CD003430

[3] WHO; FAO Diet, Nutrition and the Prevention of Chronic Diseases WHO Technical Report Series 916 WHO Geneva, Switzerland 2002

[4] Marshall JR Nutrition and colon cancer prevention Curr. Opin. Clin. Nutri. Metab Care 2009 12 539 343 10.1097/MCO.0b013e32832d6c5f 19512917

[5] Bray F Wibe A Dorum LMR Møller B Tykktarms-og endetarmskreft i Norge-epidemiologi J. Nor. Med. Assoc 2007 127 2682 2687 17952152

[6] Kirkegaard H Johnsen NF Christensen J Frederiksen K Overvad K Tjønneland A Association of adherence to lifestyle recommendations and risk of colorectal cancer: a prospective Danish cohort study BMJ 2010 341 c5504 20978063 10.1136/bmj.c5504 PMC2965150

[7] Grivennikov SI Greten FR Karin M Immunity, inflammation, and cancer Cell 2010 140 883 899 20303878 10.1016/j.cell.2010.01.025 PMC2866629

[8] Coussens LM Werb Z Inflammation and cancer Nature 2002 420 860 867 12490959 10.1038/nature01322 PMC2803035

[9] Erlinger TP Platz EA Rifai N Helzlsouer KJ C-Reactive Protein and the risk of incident colorectal cancer JAMA 2004 291 585 590 14762037 10.1001/jama.291.5.585

[10] King DE Egan BM Woolson RF Mainous AG A-Solaiman Y Jesri A Effect of a high-fiber diet *vs*. a fiber-supplemented diet on D-reactive protein level Arch. Intern. Med 2007 167 502 506 17353499 10.1001/archinte.167.5.502

[11] Ajani UA Ford ES Mokdad AH Dietary fiber and C-reactive protein: findings from national health and nutrition examination survey data J. Nutri 2004 134 1181 1184 10.1093/jn/134.5.1181 15113967

[12] Ma Y Griffith JA Chasan-Taber L Olendzki BC Jackson E Stanek EJ Li W Pagoto SL Hafner AR Ockene IS Association between dietary fiber and serum C-reactive protein Am. J. Clin. Nutri 2006 83 760 766 10.1093/ajcn/83.4.760 PMC1456807 16600925

[13] Ma Y Hebert JR Li W Bertone-Johnson ER Olendzki B Pagoto SL Tinker L Rosal MC Ockene IS Ockene JK Griffith JA Liu S Association between dietary fiber and markers of systemic inflammation in the Women’s Health Initative Observational Study Nutrition 2008 24 941 949 18562168 10.1016/j.nut.2008.04.005 PMC2603616

[14] Dunn GP Old LJ Schreiber RD The immunobiology of cancer immunosurveillance and immunoediting Immunity 2004 21 137 148 15308095 10.1016/j.immuni.2004.07.017

[15] Hong F Yan J Baran JT Allendorf DJ Hansen RD Ostroff GR Xing PX Cheung N-KV Ross GD Mechanism by which orally administered beta-1,3-glucans enhance tumoricidal activity of antitumor monoclonal antibodies in murine tumor models J. Immunol 2004 173 797 806 15240666 10.4049/jimmunol.173.2.797

[16] Cheung N-K Modak S Vickers A Knuckles B Orally administered β-glucans enhance anti-tumor effects of monoclonal antibodies Cancer Immunol. Immunother 2002 51 557 564 12384807 10.1007/s00262-002-0321-3 PMC11034228

[17] Andoh A Fujiyama Y Anti-inflammatory roles of dietary fiber and short-chain fatty acids as regards inflammatory bowel diseases Agro Food Ind Hi-Tech 2004 1 42 43

[18] Lupton JR Microbial Degradation Products Influence Colon Cancer Risk: the Butyrate Controversy J. Nutri 2004 134 479 10.1093/jn/134.2.479 14747692

[19] Bordonaro M Lazarova DL Sartorellil AC Butyraate and Wnt signaling Cell Cycle 2008 7 1178 1183 18418037 10.4161/cc.7.9.5818

[20] Volman JJ Rmakers JD Plat J Dietary modulation of immune function by β-glucans Physiol. Behav 2008 94 276 284 18222501 10.1016/j.physbeh.2007.11.045

[21] Yamada H Kiyohara H Boons G-J Lee YC Suzuki A Taniguchi N Voragen AGJ Cell Glycobiology and Development Health and Disease in Glycomedicine Elsevier Oxford, UK 2007 664 693

[22] Holtekjølen AK Uhlen AK Bråthen E Sahlstrøm S Knutsen SH Contents of starch and non-starch polysaccharides in barley varieties of different origin Food Chem 2006 94 348 358

[23] Knutsen SH Holtekjolen AK Preparation and analysis of dietary fibre constituents in whole grain from hulled and hull-less barley Food Chem 2007 102 707 715

[24] Westerlund E Andersson R Åman P Isolation and chemical characterization of water soluble mixed-linked beta-glucans and arabinoxylans in oat milling fractions Carbohydr. Polym 1993 20 115 123

[25] Misra CK Das BK Mukherjee SC Pattnaik P Effect of multiple injections of β-glucan on non-specific immune response and disease resistance in *Labeo rohita* fingerlings Fish Shellfish Immunol 2006 20 305 319 16039142 10.1016/j.fsi.2005.05.007

[26] Czop JK Austen KF Properties of glycans that activate the human alternative complement pathway and interact with the human monocyte β-glucan receptor J. Immunol 1985 135 3388 3393 4045195

[27] Akramiene D Grazeliene G Didziapetrine J Kevelaitis E Treatment of Lewis lung carcinoma by photodynamic therapy and glucan from barley Medicina (Kaunas) 2009 45 480 485 19605969

[28] Ross GD Cain JA Lachmann PJ Membrane complement receptor type three (CR_3_) has lectin-like properties analogous to bovine conglutinin and functions as a receptor for zymosan and rabbit erythrocytes as well as a receptor for iC3b J. Immunol 1985 134 3307 3315 2984286

[29] Xia Y Vetvicka V Yan J Hanikyrová M Mayadas T Ross GD The β-glucan-binding lectin site of mouse CR3 (CD11b/CD18) and its function in generationg a primed state of the receptor that mediates cytotoxic activation in response to iC3b-opsonized target cells J. Immunol 1999 162 2281 2290 9973505

[30] Thornton BP Vetvicka V Pitman M Goldman RC Ross GD Analysis of the sugar specificity and molecular location of the β-glucan-binding lectin site of complement receptor Type 3 (CD11b/CD18) J. Immunol 1996 156 1235 1246 8558003

[31] Cheung N-KV Modak S Oral (1→3),(1→4)-β-D-glucan synergizes with antiganglioside GD2 monoclonal antibody 3F8 in the therapy of neuroblastoma Clin. Cancer Res 2002 8 1217 1223 12006541

[32] Tada R Adachi Y Ishibashi K-I Tsubaki K Ohno N Binding capacity of a barley β-glucan to the β-glucan recognition molecule Dectin-1 J. Agric. Food Chem 2008 56 1442 1450 18205312 10.1021/jf073221y

[33] Tada R Ikeda F Aoki K Yoshikawa M Kato Y Adachi Y Tanioka A Ishibashi K Tsubaki K Ohno N Barley-derived β-D-glucan induces immunostimulation via a dectin-1-mediated pathway Immunol. Lett 2009 123 144 148 19428562 10.1016/j.imlet.2009.03.005

[34] Karin M Yamamoto Y Wang QM The IKK NF-kappaB system: a treasure trove for drug development Nat. Rev Drug Discovery 2004 3 17 26 10.1038/nrd1279 14708018

[35] Michaelsen TE Gilje A Samuelsen AB Høgåsen K Paulsen BS Interaction between human complement and a pectin type polysaccharide fraction, PMII, from the leaves of *Plantago major* L Scand. J. Immunol 2000 52 483 490 11119247 10.1046/j.1365-3083.2000.00801.x

[36] Di Carlo FJ Fiore JV On the composition of Zymosan Science 1958 127 756 757 13543326 10.1126/science.127.3301.756-a

[37] Soltanian S Stuyven E Cox E Sorgeloos P Bossier P Beta-glucans as immunostimulants in vertebrates and invertebrates Crit. Rev. Microbiol 2009 35 109 138 19514911 10.1080/10408410902753746

[38] Hetland G Samuelsen AB Lovik M Paulsen BS Aaberge IS Groeng EC Michaelsen TE Protective effect of *Plantago major* L. pectin polysaccharide against systemic *Streptococcus pneumoniae* infection in mice Scand. J. Immunol 2000 52 348 355 11013005 10.1046/j.1365-3083.2000.00793.x

[39] Samuelsen AB Paulsen BS Wold JK Otsuka H Kiyohara H Yamada H Knutsen SH Characterization of a biologically active pectin from *Plantago major* L Carbohydr Polym 1996 30 37 44

[40] Li Q Verma IM NF-KB regulation in the immune system Nat. Rev 2002 2 725 734 10.1038/nri910 12360211

[41] Carlsen H Moskaug JØ Fromm SH Blomhoff R *In vivo* imaging of NF-KB activity J. Immunol 2002 168 1441 1446 11801687 10.4049/jimmunol.168.3.1441

[42] Austenaa LM Carlsen H Hollung K Blomhoff HK Blomhoff R Retionoic acid dampens LPS-induced NF-κB activity: results from human monoblasts and *in vivo* imaging of NF-κB reporter mice J. Nutri. Biochem 2009 20 726 734 10.1016/j.jnutbio.2008.07.002 18926686

[43] Palma AS Feizi T Zhang Y Stoll MS Lawson AM Díaz-Rodríguez E Campanero-Rhodes MA Costa J Gordon S Brown GD Chai W Ligands for the β-glucan receptor, Dectin-1, assigned using “designer” microarrays of oligosaccharide probes (neoglycolipids) generated from glucan polysaccharides J. Biol. Chem 2006 281 5771 5779 16371356 10.1074/jbc.M511461200

[44] Izydorczyk MS Dextre JE Barley β-glucans and arabinoglucans: Molecular structure, physiochemical properties, and uses in food products—a review Food Res. Int 2008 41 850 868

[45] Holtekjølen AK Uhlen AK Bråthen E Sahlstrøm S Knutsen SH Contents of starch and son-starch polysaccharides in barley varieties of different origin Food Chem 2006 94 348 358

[46] Janeway CA Travers P Immunbiology the Immune System in Health and Disease Current Biology. Ltd London, UK 1996 31 50

[47] Roitt I Brostoff J Male D Immunology Mandarin Offset Ltd Hong Kong, China 1993

[48] Samuelsen AB Polysaccharides in *Plantago major* L. Studies of structure and biological activity Ph. D. Thesis University of Oslo Oslo, Norway 1998

[49] Moure A Gullon P Dominguez H Parajo JC Advances in the manufacture, purification and applications of xylo-oligosaccharides as food additives and nutraceuticals Process Biochem 2006 41 1913 1923

[50] Femia AP Salvadori M Broekaert WF Francois IEJA Delcour JA Courtin CM Caderni G Arabinoxylan-oligosaccharides (AXOS) reduce preneoplastic lesions in the colon of rats treated with 1,2-dimethylhydrazine (DMH) Eur. J. Nutri 2010 49 127 132 10.1007/s00394-009-0050-x 19711111

[51] Knutsen SH Holtekjølen AK Preparation and analysis of dietary fibre constituents in whole grain from hulled and hull-less barley Food Chem 2007 102 707 715

[52] Chambers RE Clamp JR An assessment of methanolysis and other factors used in the analysis of carbohydrate-containing materials J. Biochem 1971 125 1009 1018 10.1042/bj1251009 PMC1178263 5144210

[53] Samuelsen AB Paulsen BS Wold JK Otsuka H Yamada H Espevik T Isolation and partial characterization of biologically active polysaccharides from *Plantago majo*r L Phytother. Res 1995 9 211 218

[54] Vistica DT Skehan P Scudiero D Monks A Pittman A Boyd MR Tetrazolium-based Assays for Cellular Viability: A Critical Examination of Selected Parameters Affecting Formazan Production Cancer Res 1991 51 2515 2520 2021931

